# Pediatric cystine stone successfully treated by mini‐percutaneous nephrolithotripsy and antegrade ureteroscopy

**DOI:** 10.1002/iju5.12570

**Published:** 2023-01-03

**Authors:** Hiroaki Kakinoki, Yukako Yamaguchi, Yuka Kakinoki, Kazuma Udo, Shohei Tobu, Mitsuru Noguchi

**Affiliations:** ^1^ Department of Urology, Faculty of Medicine Saga University Saga Japan

**Keywords:** antegrade ureteroscopy, cystine stone, pediatric urology, percutaneous nephrolithotripsy

## Abstract

**Introduction:**

Cystinuria is often diagnosed by large renal stone for pediatric patients. The patients suffer from recurrence of stone disease, develop the chronic kidney disease and fall into end‐stage renal failure. Total removal of stone at the first intervention and prevention of recurrence are essential. Although, it is difficult to treat the pediatric stone patients for their anatomical feature.

**Case presentation:**

We report three cases of pediatric cystine stone patients (two 4‐year‐old boys and a 9‐year‐old girl) successfully treated by mini‐percutaneous nephrolithotripsy and antegrade ureteroscopy. We could remove stones completely in all three cases, and the patients did not suffer from major complications.

**Conclusion:**

It is essential to select the surgical approach, the endourological device, and the patient's position which is suitable for the age, the body size, and the condition of stones at the initial intervention of pediatric cystine stone.

**Keynote message**

Pediatric cystine stones are rare inherited disorders. The patients reveal the large volume stone and it is often difficult to remove totally. We could successfully treat three patients by mini‐percutaneous nephrolithotripsy (PNL) and antegrade ureteroscopy (URS). It is essential to select the endourological device and the patient's position.

**Abbreviations & Acronyms**
NCCT: non‐contrast‐enhanced computer tomography
PNL: percutaneous nephrolithotripsy
SWL: shock wave lithotripsy
URS: ureteroscopy

## Introduction

Cystinuria is an inherited disorder of dibasic amino acid transport system in the renal proximal tubule and the small intestine, consisting of 1% to 2% in adult urolithiasis and 10% in pediatric urolithiasis.^1^ Some cases of cystinuria were associated with chronic kidney disease and fell into end‐stage renal failure.^2^ The patients of cystinuria often reveal the large volume stone at the initial diagnosis. This is the case report of three pediatric cystine stone patients successfully treated by mini‐PNL and antegrade URS.

## Case report

### Case 1

A 4‐year‐old boy performed urethroplasty for hypospadias got fever and vomiting. He did not have family history of cystinuria. Abdominal X‐ray showed a 9 × 8 mm stone at right kidney area and a 20 × 12 mm stone at right proximal ureter area. Non‐contrast‐enhanced computer tomography (NCCT) showed a 10 × 6 mm stone at right lower calices and a 13 × 8 mm stone at right proximal ureter. He was diagnosed as obstructed pyelonephritis and the status of inflammation was improved by antibiotics. Then, we performed mini‐PNL of twice procedures (146 and 95 min, 8 days‐interval).

### Case 2

A 4‐year‐old boy with family history of cystinuria got fever and abdominal pain. Abdominal X‐ray showed a 28 × 12 mm stone at right proximal ureter area and a 15 × 10 mm at right distal ureter area. NCCT showed a 13 × 12 mm stone at right proximal ureter, a 11 × 8 mm stone at right distal ureter, right hydronephrosis, and abscess at the upper pole of right kidney. The status of inflammation was improved after antibiotics. Then, we performed antegrade URS of three times of surgeries (128, 182, and 163 min, 4 and 11 days interval).

### Case 3

**Fig. 1:**
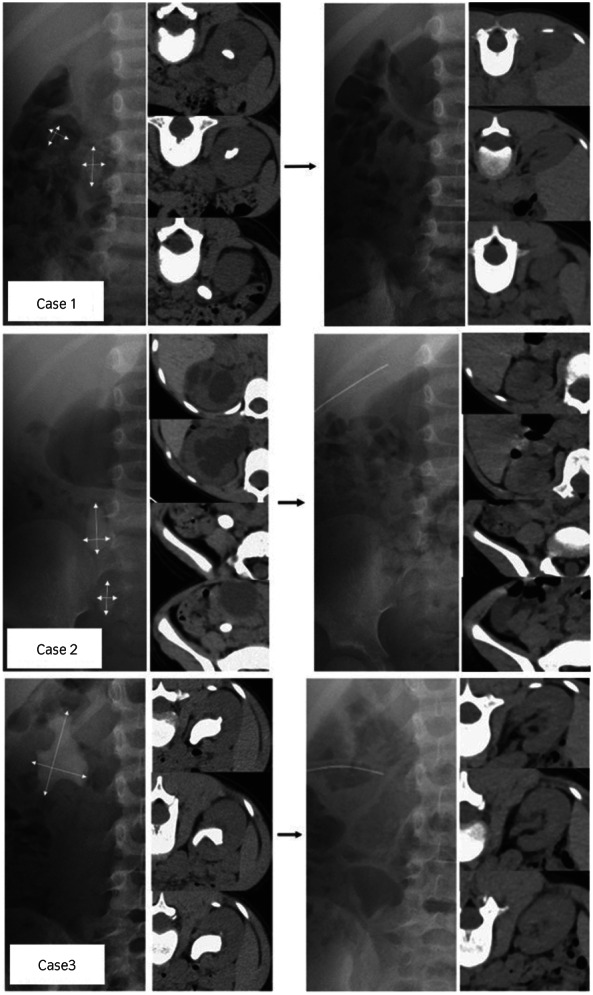
Images of all three cases before and after treatment.

A 9‐year‐old girl with family history of cystinuria was detected a staghorn stone in right kidney, which was shown 49 × 31 mm in abdominal X‐ray and a 35 × 19 mm in NCCT without symptoms. Mini‐PNL of twice procedures (134 and 163 min, 5 days interval) were performed (Fig. 1).

Patient's characteristics and laser settings of three cases are shown in table 1. The results of infrared spectroscopy were pure cystine component in all three cases. They did not suffer from major complications and have no recurrence of the stone with oral administration of the thiol drug and urine alkalization agent (follow‐up period were 60 months in case 1, 30 months in case 2, and 12 months in case 3).

**Table 1:** Patient's characteristics

Case number	Age (year)	Gender	Height (cm)	Weight (kg)	Family history of cystinuria	Position at surgery	Holmium laser setting	Follow‐up period (months)
1	4	Boy	101	15.0	Sporadic	Prone	0.8–1.2 J/10–20 Hz	60
2	4	Boy	96	14.0	Familial	Flank	0.5–0.6 J/5–10 Hz	30
3	9	Girl	122	24.5	Familial	Prone	Data were not found	12

**Fig. 2:**
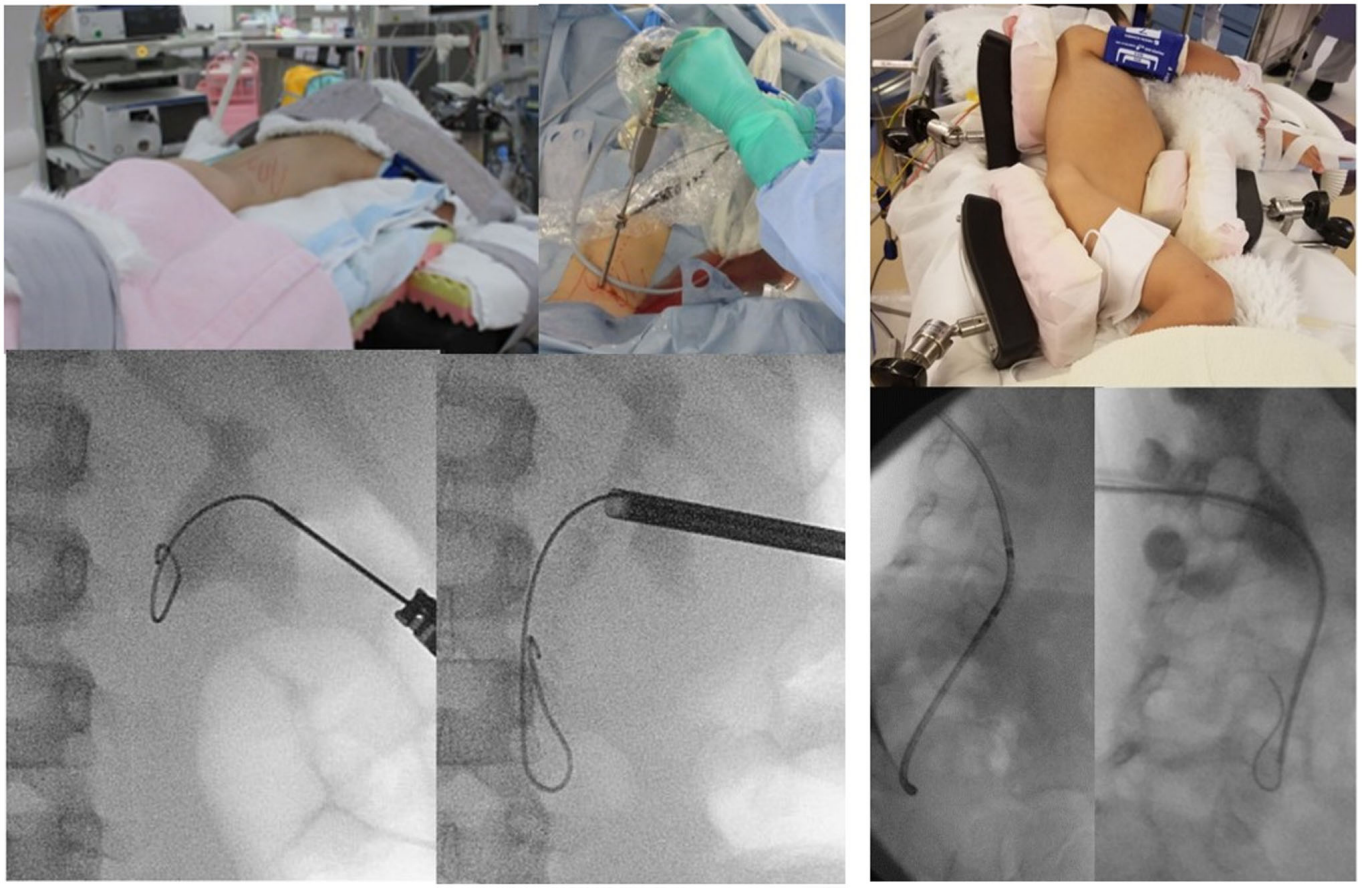
Mini‐PNL in the prone position (cases 1 and 3), and antegrade URS in the flank position (case 2).

Surgery was performed as follows. Mini‐PNL (cases 1 and 3): the patient was set in the prone position after induction of general anesthesia. The 18‐gage Chiba needle was punctured into the dorsal middle calices under the guidance of ultrasonography and fluoroscopy and a 0.035 inch guidewire was took into the collecting system. After the dilation of the access tract, the renal stone was visualized by the miniaturized nephroscopy (16 Fr outer sheath, Karl Storz) and treated by Holmium laser dusting (Versa Pulse Select 30 Lumenis). We closed the procedure about 100–180 min and a 14Fr nephrostomy tube was inserted. The fragment migrated into ureter was removed by antegrade flexible URS. Antegrade URS (case 2): the patient was set in the flank position after induction of general anesthesia. The procedure of puncture and tract dilation is same as mini‐PNL. The stones were visualized by the flexible URS (P6 or P7 4.9/7.95 Fr Olympus) through the 15 Fr outer sheath. Holmium laser lithotripsy and removal of the fragment by N Circle Basket (1.5 Fr Cook) were performed and 14 Fr Nephrostomy tube was inserted (Fig. 2).

## Discussion

Treatment of pediatric urinary stone needs high‐quality care and sophisticated surgical technique for the smaller size of the body and visceral organs. We should choose minimally invasive approach based on the patient factors (age, gender, and body size) and the stone factor (location, size, and the composition). In >2 cm stones, PNL were reported higher success rate than retrograde URS and shock wave lithotripsy (SWL).^3^, ^4^

It is advantage of the prone position that we can get large operating space for puncture, dilation of the tract and endoscopy. Case 1 was performed urethroplasty for hypospadias with urethral stenosis, so we avoided retrograde URS and SWL. case 2, retrograde URS was another option, but we considered the pressure elevation of the collecting system and obstruction by postoperative fragments caused by large stone burden. Severe hydronephrosis and dilated proximal ureter were preferable for puncture and approach. URS could be approached by both antegrade nephrostomy and retrograde urethral access. In the third procedure we performed laser lithotripsy toward fragments migrated to urinary bladder by antegrade URS with saline flush and discharge by urethral catheter. In our institution, the adult patients are often performed endoscopic combined intrarenal surgery in the modified Valdivia position, but we chose the prone and flank position to obtain the working space widely.

We could investigate some reports about Japanese patients of cystinuria. Takahashi *et al*. reported about 22 cases of long‐term follow‐up (median 160 months). Primary intervention was four SWL cases, five URS cases, and 13 PNL cases but the most patients were adults (median age: 46 years, range: 12–82 years).^5^ Inoue *et al*. reported about bilateral cystine stones of 2‐year‐old boy successfully treated by super ultra‐mini endoscopic renal surgery (sheath 8.5/9.5Fr).^6^Currently, miniaturized nephroscopy and URS, improvement of laser technology and fine retrieval instruments have revolutionized minimally invasive surgery in children.^4^

## Conclusion

We could get the stone free status for three cases of pediatric cystine stone by mini‐PNL and antegrade URS with 15–16Fr tract size. Any patients did not suffer the complications such as bleeding and septic shock. Mini‐PNL and antegrade URS are effective for large stone burden (>2 cm). It is essential to select the endourological device and the patient position for age, body size, stone burden, and stone location.

## Author contributions

**Hiroaki Kakinoki:** Conceptualization; data curation; formal analysis; investigation; writing – original draft. **Yukako Yamaguchi:** Data curation; investigation. **Yuka Kakinoki:** Writing – review and editing. **Kazuma Udo:** Writing – review and editing. **Shohei Tobu:** Writing – review and editing. **Mitsuru Noguchi:** Project administration; supervision; writing – review and editing.

## Conflict of interest

The authors declare no conflict of interest.

## Approval of the research protocol by an Institutional Reviewer Board

This study was approved by Institutional review boards, ethics committee in Faculty of Medicine, Saga University: Number is 2022‐07‐03.

## Informed consent

Informed consent was obtained.

## Registry and the Registration No. of the study/trial

Not applicable.
